# BMC medical imaging reviewer acknowledgement 2014

**DOI:** 10.1186/s12880-015-0043-6

**Published:** 2015-02-12

**Authors:** Clare Partridge

**Affiliations:** BioMed Central, Floor 6, 236 Gray’s Inn Road, London, WC1X 8HB United Kingdom

## Abstract

The editors of BMC Medical Imaging would like to thank all our reviewers who have contributed to the journal in Volume 14 (2014).

**Jawdat Abdulla**

Denmark

**Georgi Abraham**

India

**Rodney Adam**

USA

**Carmelo Aguirre**

Spain

**Toshihiro Akisue**

Japan

**Pedro Almeida**

Portugal

**Ingvild Aune**

Norway

**Oswaldo Baffa**

Brazil

**Ovidiu-Alexandru Bajenaru**

Romania

**Richard Barr**

USA

**Esther Bastiaannet**

Netherlands

**Simona Ben-Haim**

Israel

**Charlotte Bernard**

France

**Nacim Betrouni**

France

**Sotirios Bisdas**

Germany

**Piero Boraschi**

Italy

**Steven Boyd**

Canada

**Carla Caffarelli**

Italy

**Wenan Chen**

USA

**Hyungjoon Cho**

South Korea

**Haesun Choi**

USA

**Manuel Coelho-E-Silva**

Portugal

**Viviane Créteur**

Belgium

**Marco Daperno**

Italy

**Chandan Das**

India

**Søren Daugaard**

Denmark

**Abdelazeem Dawlatly**

Albania

**Donald Dengel**

USA

**Franck Diemer**

France

**David Dunaway**

UK

**Jan Engvall**

Sweden

**Ebru Ergenekon**

Turkey

**Hannu Eskola**

Finland

**Troy Farncombe**

Canada

**Sina Farsiu**

USA

**Nathalie Faye**

France

**Harvey Feigenbaum**

USA

**Martin Fiebich**

Germany

**Irene Floriani**

Italy

**Dimitrios Fotiadis**

Greece

**Jurgen Fripp**

Australia

**Shivanand Gamanagatti**

India

**Paolo Gargiulo**

Iceland

**Gerald Gartlehner**

Austria

**Yann Gavet**

France

**Venelin Gerganov**

Germany

**Rossano Girometti**

Italy

**Prabudh Goel**

India

**Xavier Golay**

UK

**Miguel Goncalves**

UK

**Ankur Goyal**

India

**Inge Groote**

Norway

**Freedom Nkhululeko Gumedze**

South Africa

**Mehdi Habibzadeh Motlagh**

Canada

**Alexander Hammers**

UK

**Bassam Hassan**

Netherlands

**Peter Hebbard**

Australia

**Soeren Hess**

Denmark

**Thomas Heyse**

Germany

**Jean-Yves Hogrel**

France

**Martin Huellner**

Switzerland

**Etsuko Imabayashi**

Japan

**Pujol Jean Louis**

France

**Soo Ji**

USA

**Sven Jonuscheit**

UK

**Yukio Kageyama**

Japan

**Ernest Nlandu Kamavuako**

Denmark

**Devasenathipathy Kandasamy**

India

**Tomokazu Kawaoka**

Japan

**Sinan Mahir Kayiran**

Turkey

**Kevin Kelly**

USA

**Farzad Khalvati**

Canada

**Sungheon Kim**

USA

**Harald Koestler**

Germany

**Norihiro Kokudo**

Japan

**Hilmar Kuehl**

Germany

**Jens Kühn**

Germany

**Ryohei Kuwatsuru**

Japan

**Kai Labusch**

Germany

**Tim Leiner**

Netherlands

**Barrett Levesque**

USA

**Florian Lietzmann**

Germany

**Xiao Liu**

USA

**Cris L. Luengo Hendriks**

Sweden

**Chi Ma**

USA

**K S Madhusudhan**

India

**Nicola Magarelli**

Italy

**Claudia Mani**

USA

**Ken Mann**

USA

**Gregory Marin**

France

**Avner Meoded**

USA

**Dorothea Miller**

Germany

**Kyung-San Min**

South Korea

**Alireza Minagar**

USA

**Yozo Mitsui**

Japan

**Subrajeet Mohapatra**

India

**Sam Mukdadi**

USA

**Dawid Murawa**

Poland

**Arnold Ng**

Australia

**Lori Nield**

USA

**Markus Pääkkönen**

Finland

**Chen Pan**

China

**Athos Patsalides**

USA

**Frederic Paycha**

France

**Talita Perciano**

USA

**Lawrence D Platt**

USA

**Stephen Price**

UK

**Emilio Puentedura**

USA

**Nelly Pustelnik**

France

**David Ralph**

UK

**Thomas Randau**

Germany

**Daniele Regge**

Italy

**Francis Ring**

UK

**Olivier Rouvière**

France

**Adriana Sampaio**

Portugal

**Yasemin Sanli**

Turkey

**Makoto Sasaki**

Japan

**Stephen Scharf**

USA

**Menno Schoonheim**

Netherlands

**Luca Maria Sconfienza**

Italy

**Anna Sedda**

Italy

**Nagabhushan Seshadri**

UK

**Sharad Shandilya**

USA

**Sara Shimoni**

Israel

**Prabhjot Singh**

India

**Chris Sistrom**

USA

**Tom Snijders**

Netherlands

**Francesco Somma**

Italy

**Modesto Sosa**

Mexico

**Tom Sutherland**

Australia

**Alberto Tagliafico**

Italy

**David Taieb**

France

**Sabina Tangaro**

Italy

**Ahmed Tawfik**

Egypt

**Matthew Teeter**

Canada

**Yogesh Thakur**

Canada

**Sree Harsha Tirumani**

USA

**Stavros Tsantis**

Greece

**Panagiotis Tsiamyrtzis**

Greece

**Kazuhiro Tsuchiya**

Japan

**Martin Ugander**

Sweden

**Lara Vera**

Italy

**B Verbist**

Netherlands

**Justin Wan**

Canada

**Qiangbin Wan**

China

**Lisheng Wang**

China

**Gareth Webster**

UK

**Friedrich Wetterling**

Ireland

**Johan Witt**

UK

**Kyoung Sook Won**

South Korea

**Alexander Wong**

Canada

**Peng Xi**

China

**Aitang Xing**

Australia

**Jian Yu**

USA

**Chun Yuan**

USA

**Xiaobing Zou**

China

